# New perspectives in human stem cell therapeutic research

**DOI:** 10.1186/1741-7015-7-29

**Published:** 2009-06-11

**Authors:** Alan Trounson

**Affiliations:** 1 California Institute for Regenerative Medicine, 210 King Street, San Francisco, CA, 94107, USA

## Abstract

Human stem cells are in evaluation in clinical stem cell trials, primarily as autologous bone marrow studies, autologous and allogenic mesenchymal stem cell trials, and some allogenic neural stem cell transplantation projects. Safety and efficacy are being addressed for a number of disease state applications. There is considerable data supporting safety of bone marrow and mesenchymal stem cell transplants but the efficacy data are variable and of mixed benefit. Mechanisms of action of many of these cells are unknown and this raises the concern of unpredictable results in the future. Nevertheless there is considerable optimism that immune suppression and anti-inflammatory properties of mesenchymal stem cells will be of benefit for many conditions such as graft versus host disease, solid organ transplants and pulmonary fibrosis. Where bone marrow and mesenchymal stem cells are being studied for heart disease, stroke and other neurodegenerative disorders, again progress is mixed and mostly without significant benefit. However, correction of multiple sclerosis, at least in the short term is encouraging. Clinical trials on the use of embryonic stem cell derivatives for spinal injury and macular degeneration are beginning and a raft of other clinical trials can be expected soon, for example, the use of neural stem cells for killing inoperable glioma and embryonic stem cells for regenerating β islet cells for diabetes. The change in attitude to embryonic stem cell research with the incoming Obama administration heralds a new co-operative environment for study and evaluation of stem cell therapies. The Californian stem cell initiative (California Institute for Regenerative Medicine) has engendered global collaboration for this new medicine that will now also be supported by the US Federal Government. The active participation of governments, academia, biotechnology, pharmaceutical companies, and private investment is a powerful consortium for advances in health.

## Commentary

There are some major changes happening in stem cell therapies that are setting a new paradigm for regenerative medicine. The opportunity to repair and regenerate tissues injured by disease and trauma is opening the way to new optimistic treatments that need to be carefully evaluated in early clinical trials. These developments are building on the successes of bone marrow hematopoietic stem cell (HSCs) transplants that have more than 30 years of patient applications in blood diseases and cancer. The bulk of applications of the new stem cell therapies follow the expected route of autologous transplants, to avoid severe immune suppression that commonly applies to allogenic cell transplantation. However, autologous therapies require the recovery of the patient's own hematopoietic, endothelial or mesenchymal stem cells by bone marrow biopsy, mobilization of bone marrow stem cell types by administration of recombinant growth factors, and isolation of multipotent stem cells from adipose tissue.

These autologous stem cell therapies are in widespread clinical trials with varying reported clinical benefits to patients. Reports on the use of granulocyte-colony stimulating factor mobilization of bone marrow stem cells and the intracoronary administration of leukapheretically isolated HSCs, show a few subjective benefits but overall no real objective benefit in refractory ischemic heart disease [1]. Some consistency in benefit in left ventricular function and remodeling has been observed in studies of intracoronary administration of the patient's own bone marrow preparations for severe myocardial infarction [2]. There is also evidence in the few comparative clinical studies available that the endothelial (CD34+) fraction of mononuclear bone marrow cells is the potent cell type responsible for vascular growth and regeneration and the clinical improvements observed in cardiac repair [3].

**Figure 1:**
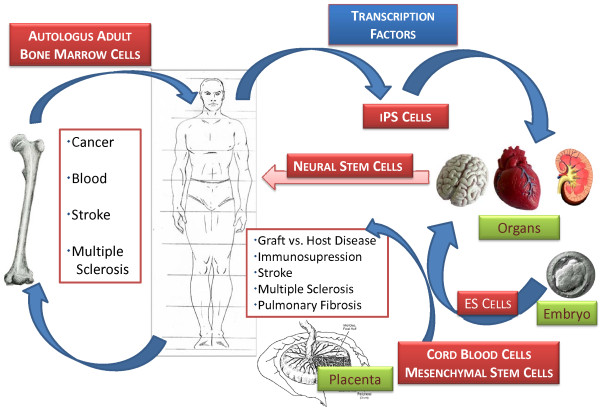
**Human stem cell transplants**. Bone marrow cells are mobilized and recovered for autologos transplantation for a number of applications, including cancer, blood diseases, stroke and multiple sclerosis. Allogenic stem cells are frequently recovered from the placenta and umbilical cord. Mesenchymal stem cells are used for immune suppression and inflammatory conditions. Neural stem cells are of fetal origin or differentiated from embryonic stem (ES) cells and induced pluripotential stem (iPS) cells. Both ES and iPS cells are poised for clinical trials for repair of spinal injury, macular degeneration, diabetes and cancer.

Aside from autologous adult stem transplants that are based mainly on the use of bone marrow, where is the field moving (Figure 1)? There is an increasing interest in allogenic stem cell therapies and advantage is being taken of cell therapies using mesenchymal (bone marrow stromal) stem cells (MSCs), which have both multipotential (by definition are capable of forming bone, adipose, and cartilage tissue), and immunosuppressive properties. The latter enables their grafting without the need for severe immunosupression and selection for histocompatibilty. These clinical trials are being led by the biotechnology companies, Osiris (Columbia, MD, USA) and Angioblast (New York, USA)/Mesoblast (Melbourne, Australia) for primary applications in preventing graft versus host disease (GvH) and treatment of cardiovascular and bone diseases. Cartilage repair using MSCs is still in preclinical and phase I studies with no detailed comparative data in humans [4]. Other very accessible sources of MSCs with apparently equivalent biological function have been identified, including the placenta [5] and Wharton's jelly of the human umbilical cord [6]. The use of cord blood cells in adults may be often accompanied by central nervous complications [7] and the need for enhancement of adaptive immunity without GvH. These conditions may be improved clinically by co-infusion of MSCs with cord blood cells [8].

Perhaps more challenging is the use of bone marrow derivatives for neural degenerative disorders. Autologous non-myeloablative HSC transplantation (mononucleocytes and platelets) has been undertaken, for example in 21 patients with relapsing-remitting multiple sclerosis to reverse neurological deficits [9]. The benefits are derived presumably from the MSC-induced immune suppression and inhibition of the proliferation of alloreactive T cells observed both *in vitro *and *in vivo *[10]. Similarly, benefits have been observed for steroid resistant GvH disease using MSCs [11,12], and also severe inflammatory diseases such as idiopathic pulmonary fibrosis [13]. While these reports are encouraging, further development and improvement of efficacy is needed because the use of MSCs for GvH and inflammatory disease is not as yet able to completely replace more conventional approaches that limit their severity [14]. Likewise, solid organ transplantation accompanied by MSC administration limits the proliferation of alloactivated T cells as shown in studies with kidney transplantation [15]. Concerns remain that while MSC administration is safe and potentially effective, the mixed results, that is, lack of demonstrated functionality and absence of long-term integration of MSC derivatives, are distinctly of issue [16].

It is also proposed that bone marrow stem cell mobilization be used to treat stroke despite little understanding of the possible mechanisms that may be involved. Clinical trials are underway and there is optimism that benefits will be clearly demonstrated [17]. Some attempts are being made to coordinate and standardize methods and assessments to enable a better determination of clinical benefit across trials, an issue that has been a constant difficulty for interpreting benefit in stem cell trials [18].

Neural stem cells (NSCs) are being explored for the treatment of lysosomal storage diseases in very young patients. The biotechnology company California Stem Cells Inc. (Irvine, CA, USA) is using transplantation of fetal NSCs for children with Batten's disease. This is an important study that addresses a very difficult genetic disease. While some benefits are reported for pharmacological enzyme replacement therapy for lysosomal storage diseases [19], long-term therapy is likely to be restricted to cell-based approaches. It is also of interest that NSCs have a remarkable tropism for malignant gliomas. Consequently, NSCs are a very likely vector to deliver oncolytic agents such as the intra-tumoral herpes simplex virus thymidine kinase [20]. There is strong interest in targeting inoperable gliobalatomas using this strategy with a range of engineered NSCs that will be seeking clinical trial approval in the near future. Some concern was raised by the report of solid multifocal brain tumor in a child with ataxia telangiectasia treated with intracerebellar and intrathecal injections of fetal NSCs in a clinic in Russia [21]. The glioneuronal neoplasm was of donor origin. There is little known of the methods used in this clinic and no reports in a large number of fetal brain tissue transplants in studies from other clinics. Nevertheless, continued vigilance in clinical trials is needed to ensure safety in all types of stem cell transplantation.

Clinical studies on the use of pluripotential stem cells are beginning. The first approved clinical trial by the Food and Drug Administration (FDA) using embryonic stem cell-derived products is for complete spinal cord injury involving low lumbar injection of glial oligodendrocytes by the biotechnology company Geron (Menlo Park, CA, USA) [22]. Their trial is in phase I testing for safety and will involve a small number of patients [23]. The International Society of Stem Cell Research has released guidelines for translational and preclinical research to ensure the global scientific community has a benchmark for the scientific and ethical principles involved in applying pluripotential stem cells for clinical treatments in regenerative medicine [24]. Many other applications are expected to enter clinical trial from embryonic stem cells (ESCs) or the new induced pluripotential stem cells (iPSCs) that are reprogrammed from mature adult cells by transduction with transcription factor DNA [25] or recombinant transcription factor proteins [26].

ESCs, and potentially iPSCs, can be directed into a wide range of cell types and proof of concept studies are taking place for preclinical studies to correct human diseases in animal models of a variety of conditions, including diabetes [27], myocardial infarction [28], Parkinson's disease [29], and liver disease [30]. One of the leading opportunities is for correction of loss of central vision through age-related macular degeneration. Human ESCs form retinal-pigmented epithelium relatively easily and research is rapidly progressing *in vitro *and *in vivo *preclinical trials. Differentiating retinal epithelial cells produce a regular, polarized monolayer with developmentally important apical and basal features consistent with developing retina, and on transplantation, rhodopsin-positive material is observed that is required of regenerating vision [31]. Regulatory approval is being sought for clinical trials following very encouraging preclinical studies in animals for the correction of blindness associated with retinal loss. Importantly, the pharmaceutical company Pfizer (Sandwich, UK) has joined the British group pioneering these studies and both Pfiza and Johnson & Johnson (Langhorne, PA) have links to the Californian biotechnology company Novocell (Irvine, CA) who are developing β islet cells derived from human ESCs for correction of diabetes. Clinical trial approval may be expected to be sought from the FDA by Novocell in the near future.

The changes that have just occurred in the US Federal Administration following the election of President Obama are substantial and important. The removal of the presidential proclamation that hindered the support of human ESC research through the US National Institutes of Health (NIH) has galvanized the scientific and biotechnology communities to accelerate progress on basic research and to stimulate translational work for regulatory approval. The Californian Institute for Regenerative Medicine (CIRM), charged with directing the US$3 billion of research funding on stem cell research, has just announced US$68 million for translational projects to complement the very substantial support of basic research, training and facilities (>US$600 million) awarded over the last 3 years. CIRM is also in the process of previewing responses for the call for preclinical 'disease team' projects that will be each funded up to US$20 million for 4 years. These projects are expected to submit for regulatory approval for clinical trials within the next 4 years. It is expected that with encouragement from CIRM, NIH, and the FDA, given the new federal administrative backing, there will be an escalation of clinical trial submissions involving pluripotent ESCs and iPSCs, multipotent placental, and adult and fetal stem cells. The challenge will remain to evaluate their effectiveness and safety, compared with any other treatments that are currently available. The need for investment for clinical trial support will be limiting unless the government and health insurance industry joins pharmaceutical and biotechnology industries to carry out the large variety of trials expected. Academic and medical research centers are also expected to be involved in the translation and clinical trial processes which heralds new opportunities for teamwork approaches for these new cell therapies.

There are also changes in interest for collaborations nationally and internationally. CIRM has collaborative agreements for joint funding of research projects with the UK, Spain, Canada, the State of Victoria in Australia, and Japan. The State of Victoria has been successful in the award of joint funding with CIRM for early translational grants, and the UK, Canada, and Spain are participating in the disease team applications. CIRM expects to work together with the NIH to promote national collaborations in stem cell research and has formulated a program of loans to support Californian companies working in stem cell science. The amalgamation of all these interests provides an extremely healthy environment for the global endeavor to deliver cell and related therapies to patients.

In conclusion, the field of stem cell sciences is rapidly integrating as new clinical trials of adult bone marrow, MSCs and NSCs for treatment of a variety of pathologies that build on more than 30 years of experience of treating blood diseases and cancer with HSCs. These new trials include many allogenic cell therapies that are more challenging than the autologous cell therapies, but within the next 10 years these will be the platform for the newly evolving pluripotential stem cell therapeutics. The latter presently involves ESCs differentiated to NSCs, glial and neuronal lineages, retinal epithelium, β islet cells, cardiomyocytes, hepatocytes, blood cells, and many other cell types and engineered tissue. Since the initial trials with patients will be phase I studies, which address safety issues, it is expected that useful clinical data on cell therapeutic effectiveness will not really be available for 5 to 10 years. While this may sound to be a long time, it is the established processes for trials that developments from discovery to clinical acceptance are generally 20 years or more. The therapies that will emerge first are likely to address neural repair, macular degeneration, cancer targeting, problems of immunity, and cardiovascular disease. However, the validity of these predictions will depend on the benefits obtained in the human phase II and III clinical trials that are still some time away. In the meantime, new candidate small molecules and bioactives are being identified using stem cell assays in high throughput screening that will impact on stem cell mobilization and expansion for regenerative medicine. Personalized medicine will also undergo a revolution with the use of iPSCs for analyzing disease heterogeneity and variable drug response.

## Abbreviations

CIRM: Californian Institute for Regenerative Medicine; ESC: embryonic stem cells; FDA: Food and Drug Administration; GvH: graft versus host disease; HSC: hematopoietic stem cell; iPSC: induced pluripotential stem cells; MSC: mesenchymal stem cells; NIH: US National Institutes of Health; NSC: neural stem cell.

## Competing interests

The author declares that they have no competing interests.

## Pre-publication history

The pre-publication history for this paper can be accessed here:


